# Protective effects on the retina after ranibizumab treatment in an ischemia model

**DOI:** 10.1371/journal.pone.0182407

**Published:** 2017-08-11

**Authors:** Stephanie C. Joachim, Marina Renner, Jacqueline Reinhard, Carsten Theiss, Caroline May, Stephanie Lohmann, Sabrina Reinehr, Gesa Stute, Andreas Faissner, Katrin Marcus, H. Burkhard Dick

**Affiliations:** 1 Experimental Eye Research, University Eye Hospital, Ruhr-University Bochum, In der Schornau 23–25, Bochum, Germany; 2 Department of Cell Morphology and Molecular Neurobiology, Faculty of Biology and Biotechnology, Ruhr-University Bochum, Universitätsstrasse 150, Bochum, Germany; 3 Department of Cytology, Faculty of Medicine, Ruhr-University Bochum, Universitätsstrasse 150, Bochum, Germany; 4 Medizinisches Proteom-Center, Ruhr-University Bochum, Universitätsstrasse 150, Bochum, Germany; University of Florida, UNITED STATES

## Abstract

Retinal ischemia is common in eye disorders, like diabetic retinopathy or retinal vascular occlusion. The goal of this study was to evaluate the potential protective effects of an intravitreally injected vascular endothelial growth factor (VEGF) inhibitor (ranibizumab) on retinal cells in an ischemia animal model via immunohistochemistry (IF) and quantitative real-time PCR (PCR). A positive binding of ranibizumab to rat VEGF-A was confirmed via dot blot. One eye underwent ischemia and a subgroup received ranibizumab. A significant VEGF increase was detected in aqueous humor of ischemic eyes (p = 0.032), whereas VEGF levels were low in ranibizumab eyes (p = 0.99). Ischemic retinas showed a significantly lower retinal ganglion cell number (RGC; IF Brn-3a: p<0.001, IF RBPMS: p<0.001; PCR: p = 0.002). The ranibizumab group displayed fewer RGCs (IF Brn-3a: 0.3, IF RBPMS: p<0.001; PCR: p = 0.007), but more than the ischemia group (IF Brn-3a: p = 0.04, IF RBPMS: p = 0.03). Photoreceptor area was decreased after ischemia (IF: p = 0.049; PCR: p = 0.511), while the ranibizumab group (IF: p = 0.947; PCR: p = 0.122) was comparable to controls. In the ischemia (p<0.001) and ranibizumab group (p<0.001) a decrease of ChAT^+^ amacrine cells was found, which was less prominent in the ranibizumab group. VEGF-receptor 2 (VEGF-R2; IF: p<0.001; PCR: p = 0.021) and macroglia (GFAP; IF: p<0.001; PCR: p<0.001) activation was present in ischemic retinas. The activation was weaker in ranibizumab retinas (VEGF-R2: IF: p = 0.1; PCR: p = 0.03; GFAP: IF: p = 0.1; PCR: p = 0.015). An increase in the number of total (IF: p = 0.003; PCR: p = 0.023) and activated microglia (IF: p<0.001; PCR: p = 0.009) was detected after ischemia. These levels were higher in the ranibizumab group (Iba1: IF: p<0.001; PCR: p = 0.018; CD68: IF: p<0.001; PCR: p = 0.004). Our findings demonstrate that photoreceptors and RGCs are protected by ranibizumab treatment. Only amacrine cells cannot be rescued. They seem to be particularly sensitive to ischemic damage and need maybe an earlier intervention.

## Introduction

Ischemia occurs during ocular diseases like age-related macular degeneration (AMD), diabetic retinopathy, central vein occlusion, or glaucoma [1–4], leading to visual impairment and possible blindness in these patients. Usually ischemia is defined by restricted blood supply to a local area, due to blockage of blood vessels leading to that area, resulting in energy depletion and cell death. In the retina, ischemia develops because of capillary blockage and leads to non-perfusion of this region. A few hours after ischemia, inflammation as well as apoptosis occurs [5].

In animal models, retinal ischemia can be induced through different techniques to study pathologic processes and explore possible treatment options. A common model is the so-called ischemia-reperfusion (I/R) animal model, where the pressure in the eye is temporarily increased through the infusion of liquid into the anterior chamber via cannulation. This leads to compression of the passing vasculature through the optic disc supplying the retina. Loss of neuronal cells, especially in the inner retinal layers, like retinal ganglion cells (RGCs) [6, 7] or amacrine cells [8, 9], is well described in this model. Previous studies indicate that these cells are most sensitive to ischemia [10, 11]. This leads to a reduced thickness of the inner retinal layers [9, 12]. Longer periods of ischemia also affect the outer retinal layers [13], including photoreceptors [14].

The vascular endothelial growth factor (VEGF) can display neurodegenerative and neuroprotective characteristics. Especially its degenerative involvement in pathological processes, such as in advanced phases of diabetic retinopathy, is still part of discussion [15]. Thus, the impact of VEGF seems to be dose-dependent. Ischemia and inflammatory events in the retina induce a VEGF response [16–18]. It is known that an upregulation of VEGF expression leads to pathological conditions, like angiogenesis, increased vascular permeability, and further inflammatory processes [19]. VEGF levels in the vitreous of diabetic retinopathy patients were reported to be elevated [20, 21], likely as a result of ischemic processes. VEGF also acts directly on different neural cell types. Therefore, it can be considered a multifunctional factor for the nervous system during development and adulthood as well asin disease conditions [22].

Initially, the primary clinical target for VEGF specific antibody treatment was cancer, but during the last years specific anti-VEGF therapies for ocular diseases were developed. Today, anti-VEGF drugs are regularly prescribed and are injected to treat retinal diseases like AMD or diabetic retinopathy [23, 24]. The three currently most common used intravitreal VEGF inhibitors are aflibercept (Eylea, Bayer), bevacizumab (Avastin, Genentech), and ranibizumab (Lucentis, Novartis). Aflibercept is a recombinant fusion protein consisting of the VEGF binding domains of human VEGF-receptors 1 and 2 fused to the human IgG_1_ Fc domain [25]. Bevacizumab is a full-length humanized murine monoclonal antibody against VEGF [26]. It has been approved by the Food and Drug Administration for intravenous treatment of certain cancers and is also used as an off-label drug to treat ocular diseases, like AMD and diabetic macular edema. Ranibizumab is a recombinant humanized monoclonal antibody fragment (Fab) that neutralizes all active forms of VEGF-A [27]. It is 10-20-fold more potent than bevacizumab in inhibiting VEGF-induced endothelial cell proliferation. Intravitreal injection of ranibizumab markedly inhibits vascularization and leakage in a primate model of laser-induced neovascularization [28]. In a current study, ranibizumab and aflibercept showed comparable ability to inhibit VEGF-induced bovine retinal microvascular endothelial cell proliferation [29]. Both had significantly greater potency than bevacizumab.

The potential neurodegenerative effect of VEGF on retinal neurons is not well investigated yet. Thus, our goal was to investigate the possible protective effect of ranibizumab treatment on retinal damage due to ischemia, animals were treated with ranibizumab three days later. Our data strongly suggest that ranibizumab is suitable to protect RGCs and photoreceptors in an ischemia-reperfusion model, while amacrine cells could not be rescued.

## Methods

### Verification of ranibizumab-binding to rat VEGF-A

The binding of ranibizumab to rat VEGF-A was verified with a self-made protein dot blot. Therefore, rat VEGF-A (Sigma-Aldrich, Taufkirchen, Germany) was resolved in 20 μl water. In total, 5 μg protein was spotted in duplicates on a black nitrocellulose-coated glass slide (Grace Bio-Labs, Bend, Oregon). Additionally, as controls, 5 μg human VEGF-A, 5 μg murine VEGF-A, 0.5 μl of the fluorophore-coupled secondary anti-IgG antibody (Alexa Fluor® 647 Goat Anti-Human IgG, Life Technologies, Darmstadt), 5 μg bevacizumab as well as 5 μg ranibizumab were spotted. Before spotting, protein concentration for all solutions was determined with amino acid analysis as described by Molina et al. [30]. The self-made protein dot blot was dried and stored until further usage as described in [31]. Protein dot blot processing and image acquisition were done as described in detail by May et al. [32]. Instead of diluted serum the dot blot was probed with a 1/50 dilution of ranibizumab in a concentration of 9.15 mg/ml.

### Animals and ethical statement

Male Brown-Norway rats were purchased from Charles River (176–200 g; Sulzfeld, Germany). The study was approved by the animal care committee of North Rhine-Westphalia (Germany) and the experiments were carried out in accordance with the ARVO statement for the use of animals in ophthalmic and vision research. Rats were housed under environmentally controlled conditions (12 h light-dark cycle) with free access to chow and water.

### Ischemia-reperfusion induction

Retinal ischemia-reperfusion (I/R) was induced as previously described [9]. Briefly, animals were anesthetized with a ketamine/xylazine/vetranquil cocktail (0.65/0.65/0.2 ml). One eye per animal was dilated with 5% tropicamide (Pharma Stulln, Stulln, Germany) and anesthetized topically with conjuncain (Bausch & Lomb, Berlin, Germany). Animals received subcutaneous carprofen injections (0.1 ml/200 g; Pfizer, Berlin, Germany), a non-steroidal anti-inflammatory drug. Intraocular pressure (IOP) was raised to 140 mm Hg for 60 min by elevating a saline reservoir connected to a 27-gauge needle (Terumo Europe, Leuven, Belgium), which was placed into the anterior chamber of one eye. Retinal ischemia was confirmed by observing whitening of the retina and reperfusion was reassured by observing the returning blood flow. The other eye remained untreated and served as a control. During the whole surgical intervention, the animals were kept on a heating pad to ensure a constant body temperature.

### Ranibizumab treatment

Three days after I/R induction, 5 μl ranibizumab (10 mg/ml; Novartis, Nürnberg, Germany) were injected intravitreally. Therefore, the animals were anesthetized with a ketamine/xylazine cocktail (100/4 mg/kg). The ischemic eye was dilated with 5% tropicamide and topically anesthetized with conjuncain. For the intravitreal ranibizumab injection, a 32-gauge Hamilton syringe (Hamilton, Reno, NV, USA) and a stereomicroscope (Carl Zeiss Microscopy, Oberkochen, Germany) were used.

### Aqueous humor and tissue collection and processing

Aqueous humor samples (n = 5-10/group) were obtained just before eye enucleation using a Hamilton syringe. Samples were stored in a freezer at -80°C until analysis. For frozen sectioning, eyes were enucleated (n = 7-10/group) and fixed in 4% paraformaldehyde, incubated in 30% sucrose and embedded in optical cutting temperature medium (Tissue-Tek; Thermo Fisher Scientific, Cheshire, UK). 10 μm retinal cross-sections were cut.

### Quantification of aqueous VEGF concentration

A colorimetric solid phase sandwich ELISA (Rat VEGF Quantikine ELISA Kit; R & D Systems, Wiesbaden-Nordenstadt, Germany) was used to determine VEGF concentrations in rat aqueous humor [33, 34]. The assay was carried out according to the manufacturer’s instructions. Measurements were performed using a Microplate Reader (AESKU Reader; AESKU.DIAGNOSTICS, Wendelsheim, Germany).

### Retina histology

Several sections per eye were stained with hematoxylin & eosin (H&E) to get a structural overview of the retinal layers. After the H&E staining, all slides were dehydrated in ethanol following incubation in xylene before being mounted with Eukitt (O-Kindler GmbH&Co, Freiburg, Germany). Two pictures of H&E stained retinal cross-sections, at a distance of 1500 μm dorsal and ventral to the optic nerve, were taken from each retina with a microscope equipped with a CCD camera (Axio Imager M1; Carl Zeiss Microscopy). The thickness of the total retina (excluding the outer segment) and ganglion cell layer (GCL) was analyzed via a measuring tool in the Zen 2012 software (Zeiss) [35]. For each analysis (total retina or GCL), three measurements per picture were prepared and then averaged.

### Immunohistology of retinal sections

Retinal cross-sections (n = 7–10 eyes/group) were prepared for immunohistochemistry. After drying and rehydration in PBS, sections were blocked in 10–20% appropriate serum with 1% BSA in 0.1% or 0.2% Triton X-100 in PBS. Three retinal sections per eye were used for each staining. RGCs, cholinergic amacrine cells, (active) microglia as well as macroglia, the synaptic ribbon terminals, photoreceptors as well as the VEGF-receptor 2 were investigated using specific antibodies (Table 1). The primary antibodies were incubated at room temperature overnight. Incubation with corresponding secondary antibodies (Table 1) was performed for 60 min. As a nuclear stain DAPI (4´,6-Diamidin-2-phenylindol; Serva Electrophoresis, Heidelberg, Germany) was added. Negative controls were implemented by using secondary antibodies only. All slides were mounted with antifade medium (Fluoro-Mount; Dianova). Four pictures per retinal cross-section, two from the periphery and two from the central part of the retina, were taken using a fluorescence microscope (Axio Imager M1 and M2; Carl Zeiss Microscopy). The pictures were taken at a distance of 300 and 3100 μm dorsal and ventral to the optic nerve, as described in a previous study of the ischemia-reperfusion model [36]. In regard to GFAP and VEGF-R2 signals, these signals were recorded with a confocal laser scanning microscope (CLSM 510; Zeiss) in combination with oil immersion lenses (Plan-Neofluor 40x/1.3Oil; Zeiss) using constant settings for each photo per stain. All digitalized images were transferred to Corel Paint Shop Photo Pro (V 13; Corel Corp., Fremont, CA, USA), masked, and excerpts were cut out.

**Table 1:** Primary and corresponding secondary antibodies used for immunohistochemistry.

Primary antibody	Company	Dilution	Secondary antibody	Company	Dilution
Anti-bassoon	Enzo life science	1:200	Donkey anti-mouse FITC	Millipore	1:500
Anti-Brn3a	Santa Cruz Biotechnology	1:100	Donkey anti-goat Alexa Flour 488	Dianova	1:500
Anti-CD68	Millipore	1:200	Goat anti-mouse Alexa Flour 488	Invitrogen	1:500
Anti-ChAT	Millipore	1:500	Donkey anti-rabbit Alexa Flour 555	Invitrogen	1:500
Anti-GFAP	Millipore	1:1000	Donkey anti-chicken Cy3	Millipore	1:700
Anti-Iba1	Wako Chemicals	1:400	Goat anti-rabbit Alexa Flour 488	Invitrogen	1:500
Anti-RBPMS	Merck	1:500	Donkey anti-rabbit Alexa Flour 488	Jackson Immuno Research	1:1500
Anti-rhodopsin	Abcam	1:500	Goat anti-mouse Alexa Fluor 488	Invitrogen	1:500
Anti-VEGF-R2	Abcam	1:100	Donkey anti-goat Alexa Flour 488	Dianova	1:500

Of each taken picture we prepared equal cut outs of a defined area (800x600 pixel; 125.14x93.86 μm). Evaluation was carried out under masked conditions with ImageJ software (V 1.44p; NIH, Bethesda, MD, USA). Brn-3a^+^, RBPMS^+^, ChAT^+^, Iba1^+^, and CD68^+^ cells were manually counted. In regard to CD68, the co-localization with Iba1 was evaluated [37]. Regarding the RGC cell counts only the Brn-3a^+^ and RBPMS^+^ cells, which were also co-localized with DAPI, were counted for the evaluation. Several research groups used these markers for RGC labeling on retinal wholemounts and cross-sections also in the ischemia-reperfusion model [38, 39]. In accordance with the published protocols and counting procedure, we used anti-Brn-3a and anti-RBPMS to label the RGC number in retinal cross-sections [40, 41].

To analyze the rhodopsin, GFAP, and VEGF-R2 staining, images were transferred in ImageJ and transformed into gray scale. After subtraction of the background (rhodopsin: 50 pixel; GFAP: 30 pixel; VEGF-R2: 30 pixel), the lower and upper thresholds were set (rhodopsin: lower threshold: 5.5, upper threshold: 260; GFAP: lower threshold: 52.35, upper threshold: 255; VEGF-R2: lower threshold: 56.01, upper threshold: 255). Background subtraction and lower and upper threshold represent mean values of all three groups. For each picture, the percentage of the rhodopsin^+^, GFAP^+^ and VEGF-R2^+^ labeled area was measured using an ImageJ macro [9, 42].

### Quantitative real-time PCR analysis of retinal tissue

For RNA isolation, retinal tissue (n = 4 eyes/group) was dissected, immediately transferred into lysis buffer with β-mercaptoethanol (Sigma-Aldrich, Steinheim, Germany) and frozen in liquid nitrogen. Total RNA was extracted and purified using the Gene Elute Mammalian Total RNA Miniprep Kit (Sigma-Aldrich). RNA concentration and purity was assessed using a BioSpectrometer® (Eppendorf, Hamburg, Germany). For reverse transcription of cDNA 1 μg RNA was used (Thermo Scientific, Waltham, MA, USA). Quantitative real-time PCR analyses were performed with Fast Start Essential DNA Green Master. For amplification, the Light Cycler® 96 (Roche Applied Science, Mannheim, Germany) was used. Oligonucleotides (Table 2) were designed using ProbeFinder software (Roche Applied Science) and ordered from Sigma-Aldrich. Primer concentration was optimized to a final concentration of 200 nM and combined with 200 ng of retinal RNAs per well. Duplicates were set for each RNA sample in a final volume of 20 μl per reaction. To obtain amplification efficiencies of different primer sets, standard curves by a two-fold dilution series with template amounts ranging from 5 to 125 ng cDNA per well were analyzed. The housekeeping gene *β-actin* was used for normalization and relative quantification.

**Table 2:** List of oligonucleotides used for mRNA expression analyses in retinas of control animals, ischemic animals, and animals treated with ranibizumab after ischemia by quantitative real-time PCR. The housekeeping gene *β-actin* served as the gene for normalization and relative quantification. The gene accession number, predicted amplicon size as well as the primer efficiency is indicated for each oligonucleotide pair. Abbreviations: bp = base pairs, F = forward, R = reverse.

Gene	Primer sequence	Amplicon size	Primer effi-ciency	GenBank accession number
*β-Actin*-F	cccgcgagtacaaccttct	72 bp	1.000	NM_031144.3
*β-Actin*-R	cgtcatccatggcgaact			
*Bassoon*-F	cctggtctcggtctggtc	65 bp	0.924	NM_019146.2, Y16563.1
*Bassoon*-R	agtctccgggaaacactgc			
*ChAT*-F	gcctcatctctggtgtgctt	62 bp	1.000	NM_001170593.1
*ChAT*-R	gtcagtgggaagggagtgg			
*CD68*-F	ctcacaaaaaggctgccact	60 bp	1.000	NM_001031638.1
*CD68*-R	ttccggtggttgtaggtgtc			
*GFAP*-F	tttctccaacctccagatcc	64 bp	0.875	NM_017009.2, L27219.1, U03700.1
*GFAP*-R	gaggtggccttctgacacag			
*Iba1*-F	ctccgaggagacgttcagtt	96 bp	0.855	NM_017196.3
*Iba1*-R	tttttctcctcatacatcagaatcat			
*Brn-3a* (*Pou4f1*)-F	ctggccaacctcaagatcc	72 bp	0.704	XM_008770931, XM_006222050
*Brn-3a* (*Pou4f1*)-R	cgtgagcgactcgaacct			
*Rhodopsin*-F	accttgagggcttctttgc	70 bp	1.000	NM_033441.1
*Rhodopsin*-R	tcaatggccaggactacca			
*VEGF*-F	actggaccctggctttactg	78 bp	0.972	BC168708.1
*VEGF*-R	tctgcttccccttctgtcgt			
*VEGF-R1*-F	cagtttccaagtggccagag	63 bp	1.000	NM_019306.2
*VEGF-R1*-R	aggtcgcgatgaatgcac			
*VEGF-R2*-F	ggagattgaaagaaggaacgag	61 bp	0.926	NM_013062.1
*VEGF-R2*-R	tggtacatttctggggtggt			

### Statistical analyses

All histological and ELISA data were transferred to a statistics and analytics software package (Statistica, V. 13.0; Dell, Tulsa, OK, USA). Histological data are presented as mean±standard error mean (SEM), ELISA data as mean±SEM±standard deviation (SD), and qRT-PCR data as median±quartile+minimum+maximum. Regarding histology and ELISA, groups were compared using ANOVA followed by Tukey post-hoc test (Dell Inc. Round Rock, TX, USA). For statistical evaluation of relative expression variations in qRT-PCR analysis, data were analyzed by REST software (QIAGEN GmbH, Hilden, Germany) using a pairwise fixed reallocation and randomization test. P-values below 0.05 were considered statistically significant.

## Results

### Positive binding capacity of ranibizumab to rat VEGF-A

**Fig 1:**
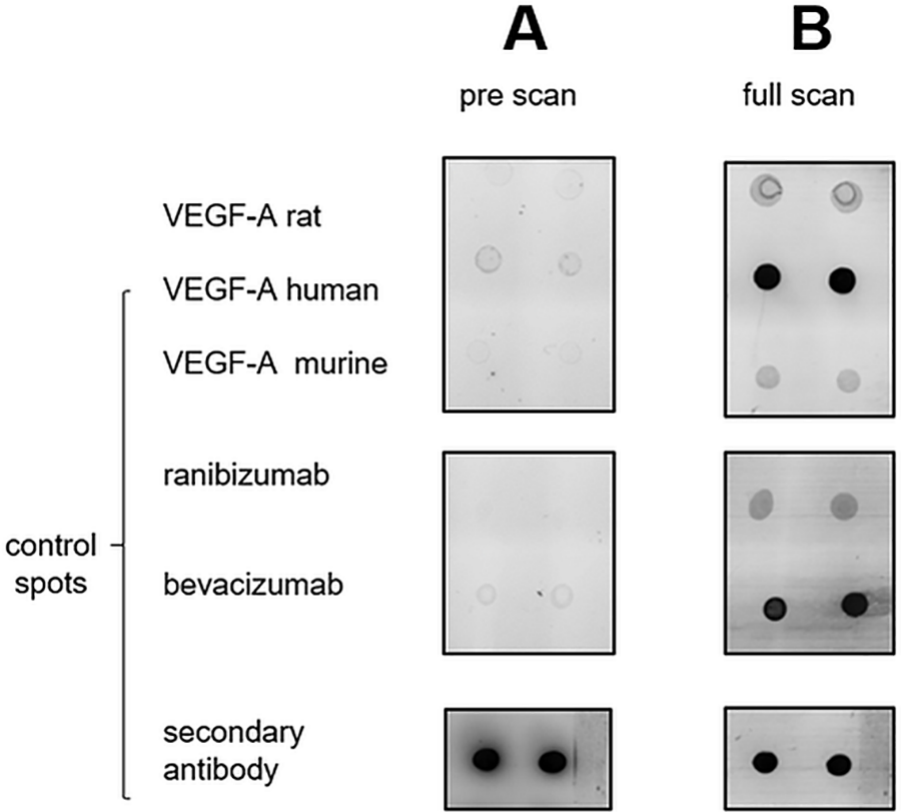
**A.** To verify the binding of ranibizumab to rat VEGF-A a protein dot blot was used. Rat VEGF-A was spotted on a nitrocellulose coated-glass slide. Human VEGF-A, murine VEGF-A, ranibizumab, bevacizumab as well as the fluorophore-coupled anti-IgG secondary antibody served as positive control. Before incubation with ranibizumab, the dot blot was scanned to exclude possible unspecific fluorescence signals. As expected, a fluorescence signal was only detected for fluorophore-coupled secondary antibody. **B.** After incubation with ranibizumab and incubation with the secondary anti-IgG antibody, fluorescence signals were detected for all spots in comparison to the pre-scan. A positive binding of ranibizumab to rat VEGF-A and not only to human VEGF-A is proven.

For verification of ranibizumab-binding to rat VEGF-A a protein dot blot based strategy was applied. Before processing the self-made dot plot, as expected, it showed only a positive fluorescence signal for the fluorophore-coupled secondary anti-IgG antibody. No signals were detected for rat, human and murine VEGF-A as well as for ranibizumab and bevacizumab (Fig 1A). After incubation with ranibizumab, positive fluorescence signals were detected for each species-specific VEGF-A as well as for ranibizumab and bevacizumab (Fig 1B). The highest fluorescence intensity among the different VEGF-A was observed for human VEGF-A. For rat and murine VEGF-A the fluorescence signal was weaker, but comparable to the ranibizumab control spots and therewith also positive for binding to ranibizumab.

### Strong influence of ischemia-reperfusion on VEGF level and retinal layers

**Fig 2:**
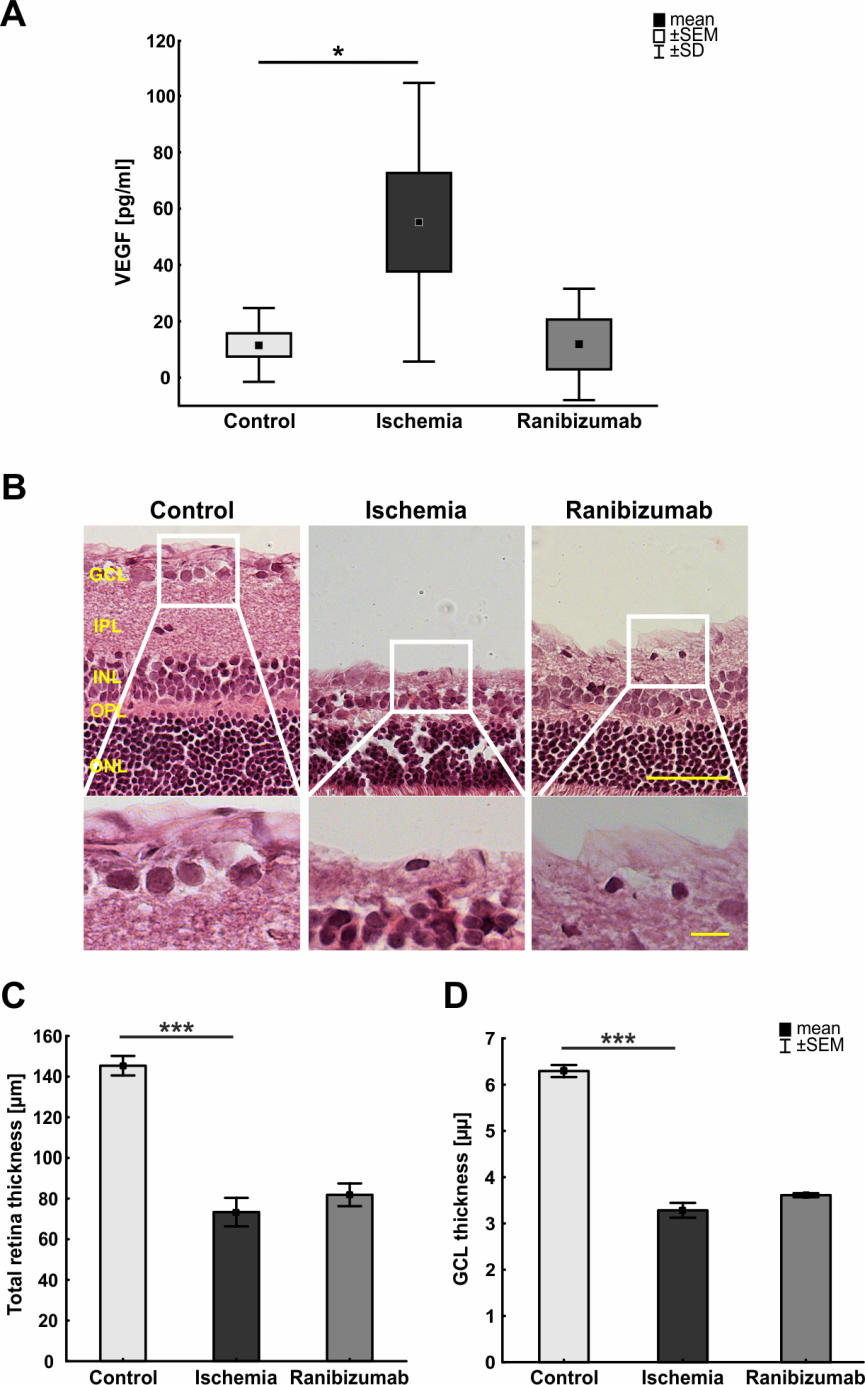
**A.** Aqueous humor VEGF levels in control animals, ischemic animals, and animals treated with ranibizumab. Aqueous humor samples were collected 21 days after ischemia. A significant increase in VEGF was observed in ischemic animals compared to controls (p = 0.032). The ranibizumab and the control groups had comparable VEGF levels (p = 0.99). **B.** Exemplary H&E stained retinal cross-sections from a control, an ischemia and a ranibizumab animal with cut outs focused on the GCL. **C.** A significantly reduced thickness of the total retina was detected in ischemic retinas compared to controls (p<0.001). Ranibizumab treated eyes showed similar reduction in retinal thickness like the ischemic eyes (p = 0.6). **D**. The measurements for the thickness of the GCL were comparable to the ones for the total retina. In ischemic retinas, a significant GCL reduction was noted in comparison to the control group (p<0.001). No significant differences could be detected between ischemic and ranibizumab treated eyes (p = 0.189). *: p< 0.05. Abbreviations: GCL = ganglion cell layer; IPL = inner plexiform layer; INL = inner nuclear layer; OPL = outer plexiform layer; ONL = outer nuclear layer; PR = photoreceptor layer. Scale bar: 50 μm (cut outs 10 μm).

A significant increase of aqueous humor VEGF levels was detected in rats undergoing ischemia (55.2±17.5 pg/ml; p = 0.032; Fig 2A). The VEGF levels of animals treated with ranibizumab after ischemia (11.8±8.8 pg/ml) were comparable to control values (11.6±4.1 pg/ml; p = 0.99) at 21 days.

An obvious decrease in thickness of some retinal layers could be observed by analysis of H&E stained retinal cross-sections 21 days after ischemia (Fig 2B). Statistical analysis revealed a significant reduced thickness of the total retina and the GCL in ischemic retinas when compared to the control groups (total and GCL: p<0.001). The reduction of retinal thickness of ranibizumab treated eyes and ischemic ones was comparable (total: p = 0.6; GCL: p = 0.189; Fig 2C and 2D). This is in accordance with previous studies by other research groups and ours, where a thinning of retinal layers was observed after ischemia [9, 43].

### Protection of retinal ganglion cells from ischemic damage through ranibizumab treatment

**Fig 3:**
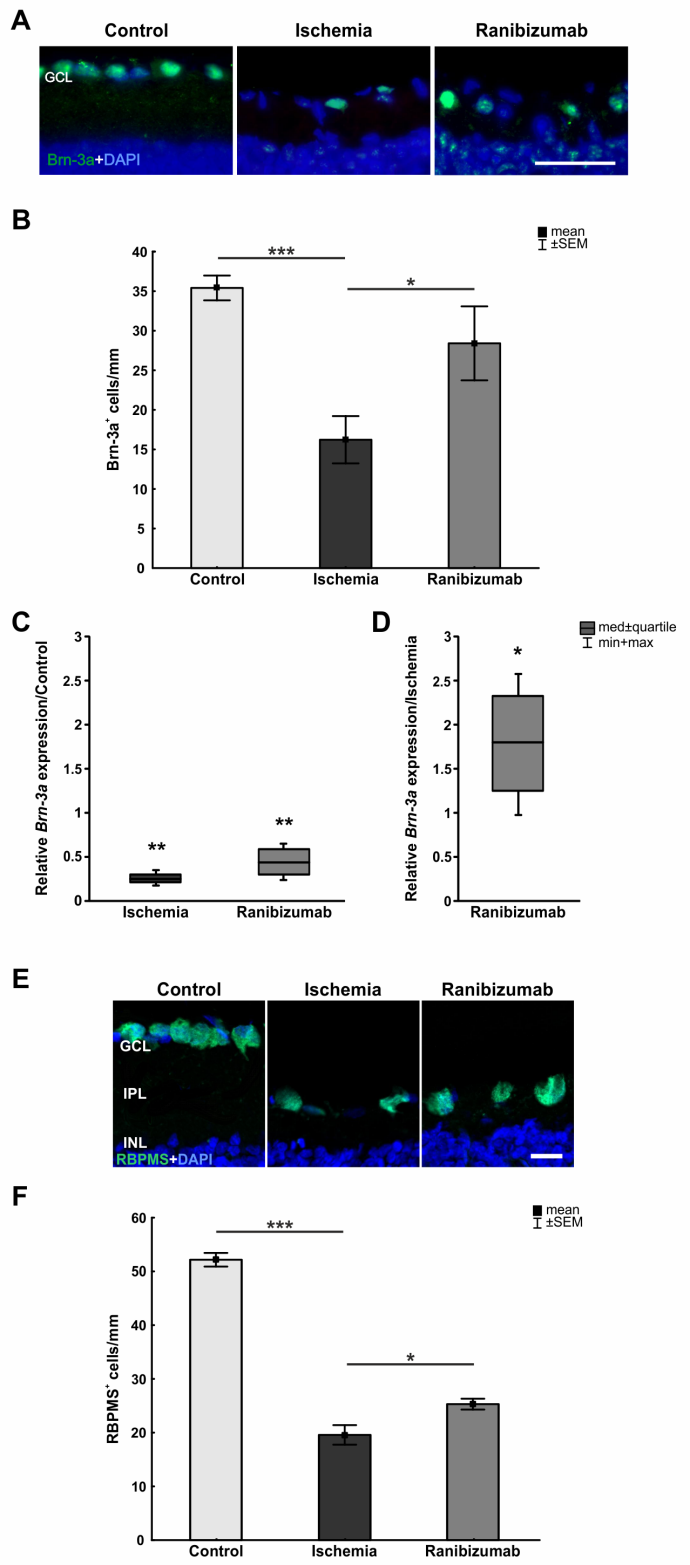
**A.** Representative retinal cross-sections of the three groups stained with anti-Brn-3a for RGCs and DAPI for cell nuclei. Fewer Brn-3a^+^ cells were observed in ischemic retinas. **B.** A significant RGC loss was noted in the ischemia group compared with control eyes (p<0.001). In comparison to the ischemic group the ranibizumab treated eyes showed a significant higher number of Brn-3a^+^ cells (p = 0.042). **C.** Via qRT-PCR a significant decrease of *Brn-3a* mRNA (RGCs) was detected in ischemic (p = 0.002) and ranibizumab treated retinas (p = 0.007). **D.** Compared to the ischemia group *Brn-3a* expression was significantly elevated in the ranibizumab group (p = 0.039). **E.** RGCs were additionally stained with the specific marker RBPMS. DAPI was used to label the cell nuclei. All ischemic retinas showed fewer RBPMS^+^ cells in the GCL. **F.** Compared to controls, a significant RBPMS^+^ RGC loss could be detected in the ischemia group (p = 0.001). Ranibizumab treated retinas also displayed significantly fewer RBPMS^+^ RGCs, when compared to controls (p = 0.001). In contrast, the ranibizumab group displayed significantly more RGCs than the ischemia group (p = 0.027). *: p<0.05; **: p<0.01; ***: p<0.001. Abbreviation: GCL = ganglion cell layer. Scale bar: 20 μm.

An antibody specific for Brn-3a was used to visualize RGCs. 21 days after ischemia fewer Brn-3a^+^ RGCs were seen in these retinas, while staining intensity in the ranibizumab group seemed to be comparable to controls (Fig 3A). Statistical analysis revealed a significant RGC loss in the ischemia group (16.2±3.0 cells/mm) compared to the control group (35.4±1.6 cells/mm; p<0.001), while no significant decrease was detected in the ranibizumab group (28.4±4.7 cells/mm; p = 0.3; Fig 3B). In comparison to the ischemia group, more RGCs were detected in the ranibizumab group (p = 0.04). In order to confirm the histological results on mRNA level, *Brn-3a* (*POU4f1)* expression was analyzed via qRT-PCR. A significant down-regulation of relative *Brn-3a* expression could be measured in ischemic (0.246 expression ratio; p = 0.002) and ranibizumab treated retinas (0.444 expression ratio; p = 0.007) compared to control ones (Fig 3C). In relation to the ischemia group, the *Brn-3a* mRNA level was significantly increased in the ranibizumab group (1.804 expression ratio; p = 0.039; Fig 3D).

RGCs were additionally visualized with anti-RBPMS antibody. This staining confirmed previous findings using anti-Brn-3a. All ischemic retinas showed fewer RBPMS^+^ cells in the GCL. A significant RGC loss could be demonstrated in the ischemia group (p<0.001) in comparison to the control. Ranibizumab treated retinas showed significantly more RBPMS^+^ RGCs when compared to the ischemia group (p = 0.027; Fig 3F).

### Loss of amacrine cells after ischemia-reperfusion with and without ranibizumab treatment

**Fig 4:**
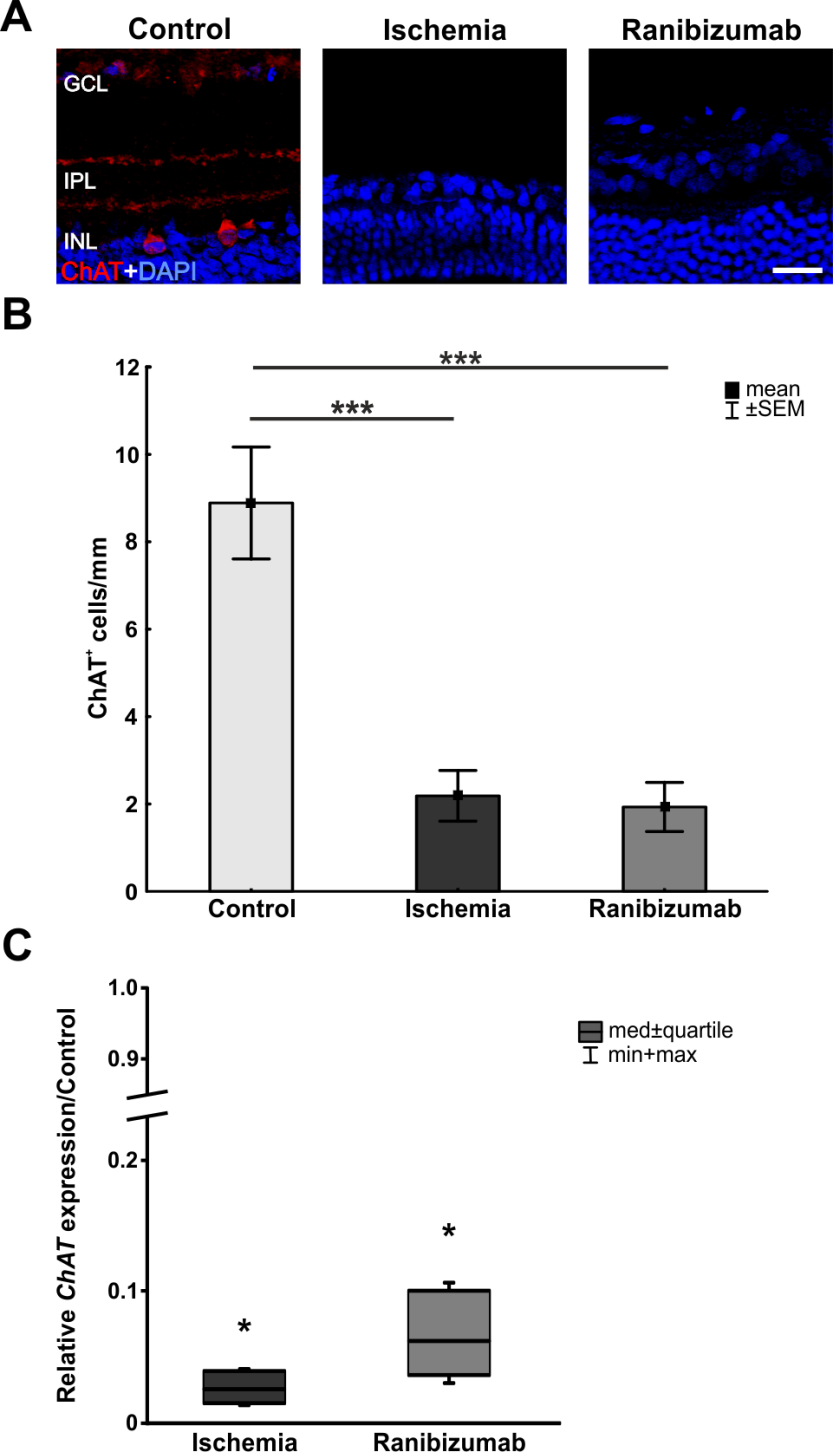
**A.** Cholinergic amacrine cells were labelled using anti-ChAT antibody. The distinct stratification and ChAT^+^ cells in the INL were observed in the control group, while both was not present in the ischemia and the ranibizumab groups. **B.** Also, in both groups fewer ChAT^+^ cells were visible (p<0.001). **C.** A significant decrease of *ChAT* mRNA was noted in the ischemia (p = 0.017) as well as in the ranibizumab groups (p = 0.014). Whereas the decrease in ranibizumab treated eyes was less prominent than in the ischemia group. There was a trend to a higher *ChAT* mRNA level in the ranibizumab group when compared to the ischemia group (p = 0.063). *: p<0.05; ***: p<0.001. Abbreviations: GCL = ganglion cell layer; IPL = inner plexiform layer; INL = inner nuclear layer. Scale bar: 20 μm.

Cholinergic amacrine cells were visualized using an anti-ChAT antibody. Sections of ischemic and ranibizumab retinas had only few ChAT^+^ immunoreactivity. Only in the control group, ChAT immunoreactivity appeared as two clearly defined synaptic strata in the inner plexiform layer (IPL) with cell bodies in the inner nuclear layer (INL; Fig 4A). Counts revealed only few ChAT^+^ cells in the ischemia (2.2±0.6 cells/mm; p<0.001) and the ranibizumab groups (1.9±0.6 cells/mm; p<0.001), when compared to controls (8.9±1.3 cells/mm; Fig 4B). In regard to the relative *ChAT* expression, a significant decrease in the mRNA level was detected in ischemic (0.025 expression ratio; p = 0.017) and in ranibizumab treated retinas (0.062 expression ratio; p = 0.014; Fig 4C). The down-regulation of *ChAT* mRNA level in the ranibizumab group was less intense than in ischemic retinas, (p = 0.063; data not shown).

### A certain preservation of photoreceptor cells after ischemia-reperfusion with ranibizumab treatment

**Fig 5:**
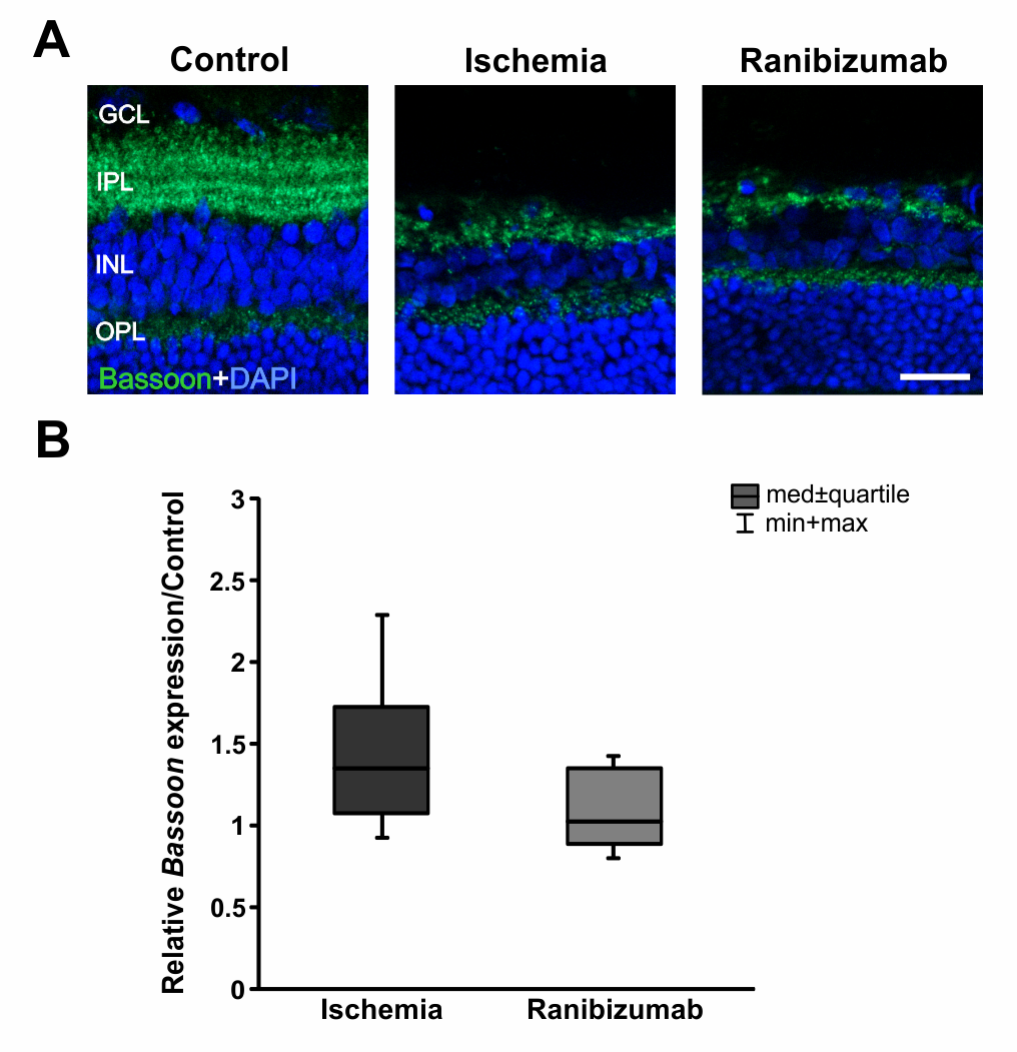
**A.** Ribbon terminals were marked with pre-synaptic bassoon. Intense staining and distinct stratification were noted in the IPL, mainly in retinas of control eyes. **B.** No differences could be measured in regard to *bassoon* mRNA levels in both, the ischemic group (p = 0.077) and the ranibizumab group (p = 0.892), when compared to controls. Abbreviations: GCL = ganglion cell layer; IPL = inner plexiform layer; INL = inner nuclear layer; OPL = outer plexiform layer. Scale bar: 20 μm.

Photoreceptor synaptic ribbon terminals in the outer plexiform layer (OPL) and amacrine synapses in the IPL were marked using an antibody specific for the presynaptic protein bassoon. A decline in bassoon immunoreactive presynaptic puncta was visible in ischemic and ranibizumab eyes (Fig 5A). Control eyes displayed a prominent immunoreactivity of bassoon with distinct strata in the IPL. On mRNA level, no differences could be detected in *bassoon* mRNA expression levels in the ischemia (1.349 expression ratio; p = 0.077) or the ranibizumab treated group (1.022 expression ratio; p = 0.892) compared to controls (Fig 5B). Even though, *bassoon* mRNA expression was less expressed in ranibizumab eyes in comparison to ischemic ones (p = 0.023; data not shown).

**Fig 6:**
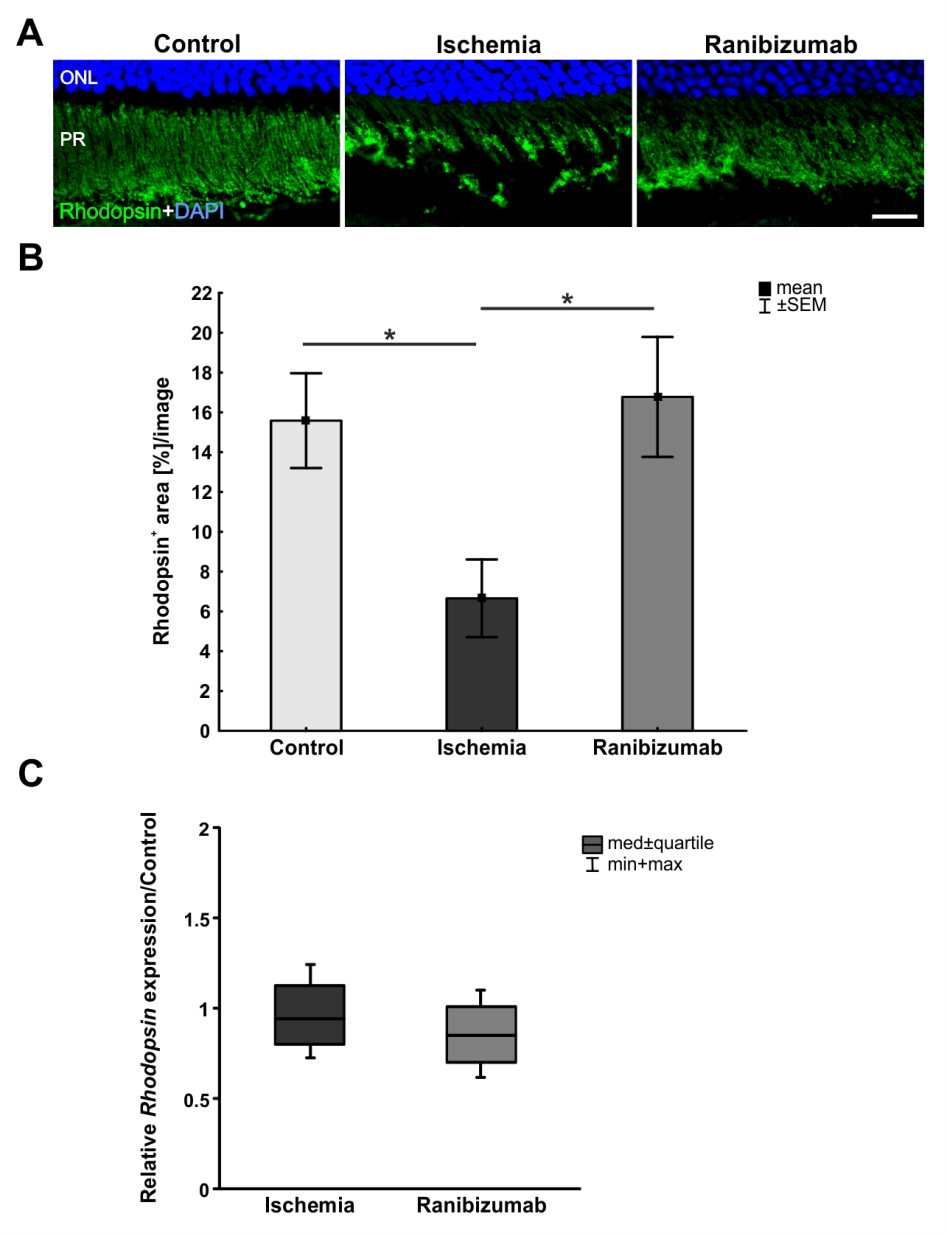
**A.** Rhodopsin was used to stain rod photoreceptors of control, ischemic and ranibizumab treated eyes. In the control group, rod photoreceptors were clearly visible and their structure was well defined. After ischemic injury, the structure appeared disordered and damaged, while after treatment with ranibizumab it seemed to be more organized. **B.** Compared to controls, the ischemia group showed a significant reduced rhodopsin^+^ area (p = 0.049). In the ranibizumab group, a significant enhanced rhodopsin^+^ area was noted in relation to the ischemia group (p = 0.036). **C.** On mRNA level, no differences could be measured in ischemic retinas (p = 0.511) or ranibizumab treated ones (p = 0.122) in comparison to controls. *: p<0.05. Abbreviations: ONL = outer nuclear layer; PR = photoreceptors. Scale bar: 20 μm.

Anti-rhodopsin staining was used to visualize photoreceptor rods (Fig 6A). Area analysis showed a decrease in rhodopsin^+^ staining area in ischemic eyes (6.7±2.0% area/image) compared to controls (15.6±2.4% area/image; p = 0.049; Fig 6B). No significant differences were detected between control and ranibizumab eyes (16.8±3.0% area/image; p = 0.947) in regard to rhodopsin^+^ area. The relative *rhodopsin* mRNA expression was analyzed via qRT-PCR. Here, no differences were noted in the ischemia (0.94 expression ratio; p = 0.511) and the ranibizumab groups (0.851 expression ratio; p = 0.122) compared to controls (Fig 6C). Also, no alterations could be observed regarding *rhodopsin* mRNA expression levels in ranibizumab treated eyes compared to the ischemia group (p = 0.426; data not shown).

### Lower expression of macroglia and VEGF-receptor 2 after ischemia-reperfusion with ranibizumab treatment

**Fig 7:**
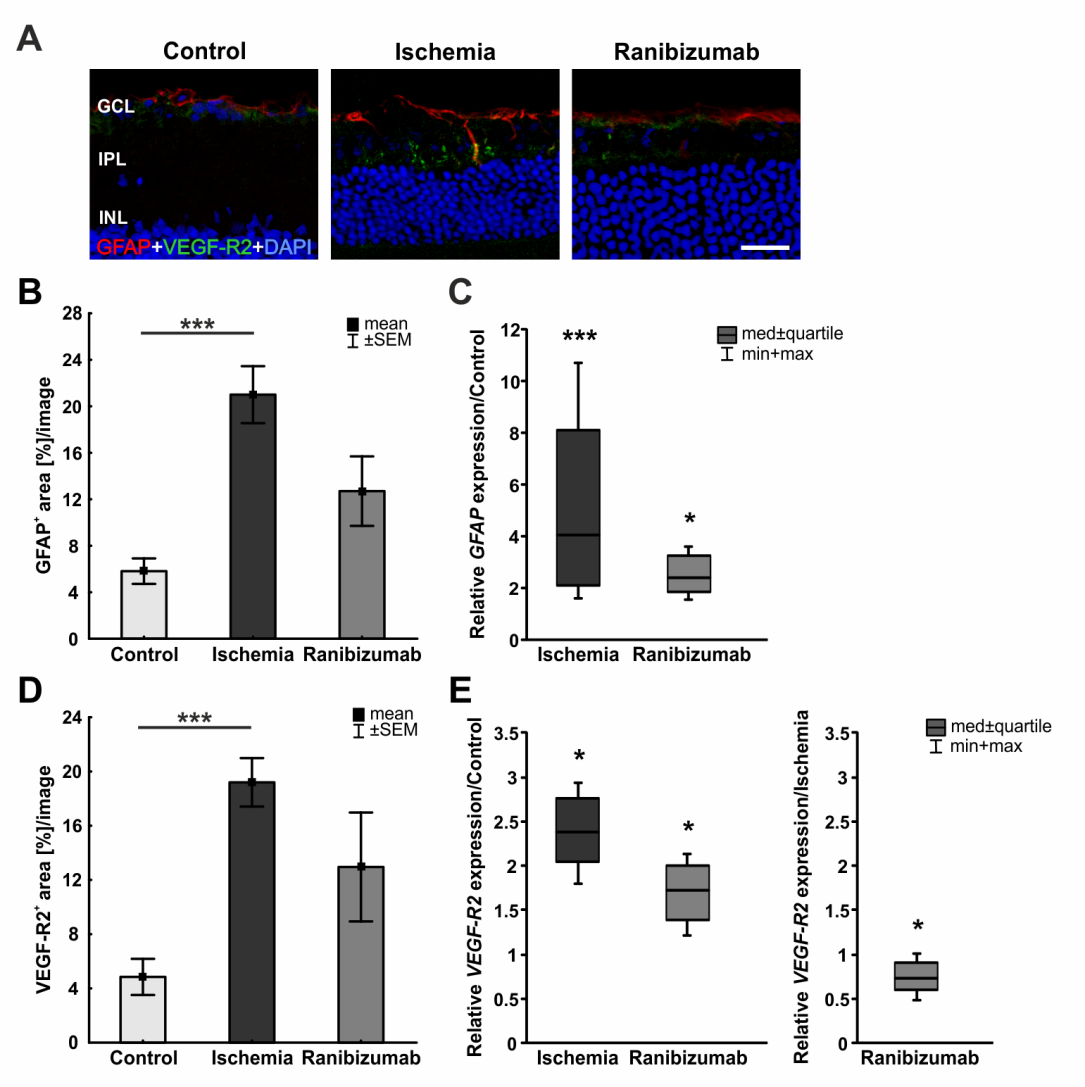
**A.** Exemplary retinal sections labelled with anti-GFAP (red) and anti-VEGF-R2 (green). **B.** GFAP^+^ area was significantly increased in ischemic retinas (p<0.001), while it was less prominent in ranibizumab ones (p = 0.1). **C.** On mRNA level, a significant up-regulation in *GFAP* expression could be shown in the ischemia (p<0.001) as well as in the ranibizumab groups (p = 0.015) in relation to controls. **D.** Ischemic retinas displayed a significantly larger VEGF-R2^+^ area than controls (p = 0.0009). Also, a trend in ranibizumab treated eyes could be noted (p = 0.1). **E.** Compared to controls, a significant increase in *VEGF-R2* mRNA expression was measured in ischemic (p = 0.021) and ranibizumab treated eyes (p = 0.03). Whereas *VEGF-R2* mRNA level was significantly lower in the ranibizumab group than in the ischemia group (p = 0.046). ***: p<0.001. Abbreviations: GCL = ganglion cell layer; IPL = inner plexiform layer; INL = inner nuclear layer. Scale bar: 20 μm.

In order to visualize macroglia and VEGF-receptor 2 expression, anti-GFAP and anti-VEGF-R2 antibodies were applied to retinal sections. GFAP is mainly expressed by astrocytes, the labelling was localized in close proximity to the GCL of all groups. In the ischemia and the ranibizumab groups, processes of radially oriented Müller cells also displayed GFAP-positive immunoreactivity (Fig 7A). In regard to VEGF-R2 staining, only minor signals were observed on control retinas, while a scattered VEGF-R2 signal was noted in ischemic and ranibizumab retinas, mainly in the IPL. A larger immunopositive GFAP signal area was noted in the ischemic group (21.0±2.5% area/image) in comparison to controls (5.8±1.1% area/image; p<0.001). The ranibizumab group (12.7±3.0% area/image) displayed a trend towards a larger GFAP staining area (p = 0.1; Fig 7B). Anti-VEGF-R2^+^ staining area was significantly increased in the ischemic group (19.2±1.8% area/image) compared to the control group (4.8±1.3% area/image; p<0.001). Again, the ranibizumab group (13.0±4.0% area/image) displayed a trend towards an increased VEGF-R2^+^ staining area (p = 0.1; Fig 7D). In addition, the relative *GFAP* and *VEGF-R2* mRNA expression were analyzed via qRT-PCR. A significant up-regulation of *GFAP* mRNA could be measured in the ischemic group (4.036 expression ratio; p<0.001) and the ranibizumab group (2.403 expression ratio; p = 0.015) in comparison to controls (Fig 7C). However, the *GFAP* mRNA increase in ranibizumab treated eyes was less prominent than in ischemic ones. No differences were noted in regard to *GFAP* mRNA levels in the ranibizumab group compared to the ischemia group (p = 0.238; data not shown). Also, *VEGF-R2* mRNA expression was significantly increased in ischemic (2.381 expression ratio; p = 0.021) and in ranibizumab retinas (1.727 expression ratio; p = 0.03; Fig 7E). In relation to the ischemic group the *VEGF-R2* mRNA expression was significantly lower in the ranibizumab group (0.725 expression ratio; p = 0.046) in comparison to the ischemic group (Fig 7E). Additionally, the *VEGF* and *VEGF-R1* expression was measured via qRT-PCR. No differences were noted between the ischemia group and the control group (p = 0.188) in regard to *VEGF* mRNA expression levels. A significant decrease of *VEGF* mRNA expression was observed in ranibizumab treated eyes in comparison to control retinas (p = 0.003; data not shown). No changes in *VEGF-R1* mRNA level were observed in ischemic (p = 0.119) and ranibizumab treated eyes (p = 0.972) compared to controls (data not shown).

### Increased numbers of microglia after ischemia-reperfusion with and without ranibizumab treatment

**Fig 8:**
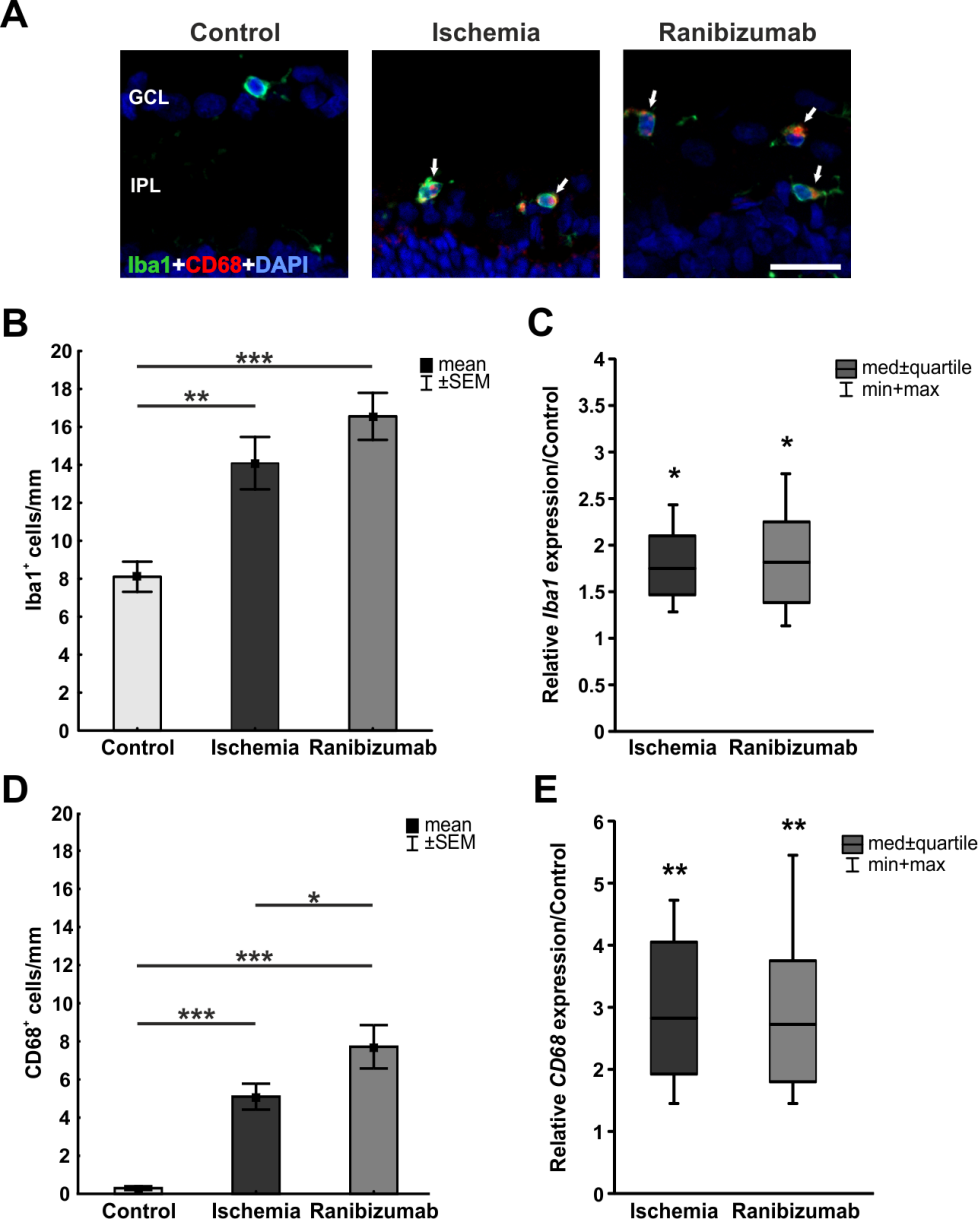
**A.** Microglia cells were labeled with Iba1 (green), while activated microglia and macrophages were detected with CD68 (red). In all three groups, the microglia as well as their activated type were present in the GCL, IPL, and INL. CD68^+^ cells could only be noted in ischemic and ranibizumab eyes. **B.** Counting of Iba1^+^ cells revealed a significant increase in cell number in the ischemia (p = 0.003) and the ranibizumab groups (p<0.001) compared to controls. **C.** Also, via qRT-PCR a significant increase of *Iba1* mRNA expression was observed in ischemic (p = 0.023) and ranibizumab eyes (p = 0.018) in comparison to controls. **D.** Additionally, a significant increase of activated microglia and macrophages (CD68) could be observed in the ischemia (p<0.001) and the ranibizumab groups (p<0.001). **E.** Equally, *CD68* mRNA (activated microglia) expression was significantly up-regulated in ischemic (p = 0.009) and ranibizumab retinas (p = 0.004) in relation to controls. *: p<0.05; **: p<0.01; ***: p<0.001. Abbreviations: GCL = ganglion cell layer; IPL = inner plexiform layer. Scale bar: 20 μm.

Microglia were visualized using an anti-Iba1 antibody, while an anti-CD68 antibody was used to specifically label activated ones. A lot more microglia were noted in ischemic and ranibizumab retinas, many of them were activated (Fig 8A). Cell counts confirmed this observation. Significantly higher numbers of Iba1^+^ microglia were noted in ischemic (14.1±1.4 cells/mm; p = 0.003) and ranibizumab retinas (16.6±1.2 cells/mm; p<0.001) compared to controls (8.1±0.8 cells/mm; Fig 8B). In addition, more activated CD68^+^ microglia were detected in the ischemia (5.1±0.7 cells/mm; p<0.001) and the ranibizumab groups (7.7±1.1 cells/mm; p<0.001) when compared to the control group (0.3±0.1 cells/mm; Fig 8D). The total number of microglia and the number of activated ones was slightly higher in the ranibizumab group than in the ischemia group. In accordance with the immunohistological data, a significant higher *Iba1* mRNA expression was detected in ischemic (1.746 expression ratio; p = 0.023) and ranibizumab treated eyes (1.824 expression ratio; p = 0.018) when compared to controls (Fig 8C). There was even a trend towards a higher *Iba1* mRNA expression in ranibizumab treated eyes in comparison to ischemic ones (p = 0.056; data not shown). Equally, *CD68* mRNA (activated microglia) expression was significantly increased in the ischemia (2.824 expression ratio; p = 0.009) and in the ranibizumab groups (2.742 expression ratio; p = 0.004) in relation to controls (Fig 8E).

## Discussion

Ischemia-reperfusion injury affects O_2_-dependent cells of tissues and organs, such as heart, brain, liver, kidney, intestine, or retina [44]. It is a major reason for organ dysfunction and non-function following transplantation. Retinal ischemia is a result of pathologic processes that damage retinal vessels [45]. Severe retinal ischemia leads to retinal neovascularization. These diseases are referred to as ischemic retinopathies and include diabetic retinopathy, retinal vein occlusions, or retinopathy of prematurity. Unfortunately, the role of retinal ischemia in those diseases is not yet understood in detail. I/R is therefore a common model to study possible protective agents against retinal degeneration.

We investigated the possible effect of intravitreal treatment with the anti-VEGF antibody ranibizumab after retinal ischemia. Our results demonstrate that intravitreal VEGF injection three days after I/R leads to a certain protection of neuronal cells, like RGCs and photoreceptors. Additionally, macroglia response was less pronounced after ranibizumab treatment, while an increase in microglia was noted.

In this study, we observed increased aqueous humor VEGF levels in animals that underwent ischemia. In a recent study by Kovacs et al. significantly elevated levels of growth factors, such as VEGF, were measured in patients with retinal ischemia [46]. Increased VEGF levels were detected in diabetes patients with or without proliferative diabetic retinopathy and in patients with neovascular glaucoma.

VEGF is found in all major classes of retinal neurons in the human retina. Labelling could be specially noted in RGCs and in amacrine cells [47]. Also, in the rat retina VEGF is predominantly localized to neurons and scarcely to retinal vessels. VEGF immunoreactivity can be observed in the GCL and INL [48]. It is known that retinal VEGF is affected by ischemia [16] and its expression in neurons is increased in a retinal ischemia animal model [49]. The retina seems to respond to ischemic damage with a rapidly increased production of angiogenic factors. Thus, VEGF levels are already elevated after 24 hours.

Neuroprotective properties of VEGF, but also its neurodegenerative ones, are still part of discussion. VEGF also acts directly on different neural cell types. Therefore, it can be considered a multifunctional factor for the nervous system during development and adulthood and in disease conditions [22]. In Multiple Sclerosis (MS), VEGF might aggravate disease progression. In EAE, a MS animal model, intracerebral application of VEGF resulted in the development of massive inflammatory reactions [50]. The authors found VEGF to be upregulated in MS plaques and EAE lesions. This study suggests that blockage of brain endothelial cell VEGF signaling might be a protective avenue in patients with MS. The neurodegenerative effect of VEGF on retinal neurons is not well investigated[15]. Tolentino et al. detected a retinal microangiopathy and ischemia after intravitreal injection of VEGF in non-human primates [51]. In a rat model of cerebral ischemia, Zhang et al. could demonstrate that VEGF leads to a disturbance of the blood-brain barrier with increased permeability and increased ischemic lesions [52].

Müller cells do express the VEGF receptors VEGF-R1 and VEGF-R2. These cells undergo apoptosis in mice with systemic VEGF neutralization [53]. Glial and neuronal cells require VEGF as a survival factor under normal conditions [48]. In a primary rat retina culture treatment with ranibizumab induced an activation of Müller glia [54]. A typical response of gliotic Müller cells includes an increased expression of the intermediate filament GFAP. Healthy rabbits receiving repeated intravitreal injection of ranibizumab, displayed an increase in GFAP immunoreactivity, which was mainly localized in astrocytes [55]. In our study, retinal ischemia leads to an activation of macroglia on mRNA and protein level. It has been suggested that Müller cells and astrocytes undergo gliosis following ischemia [56]. Animals additionally treated with ranibizumab also showed increased levels of *GFAP* mRNA, although not as prominent as ischemic retinas. A trend to an increase in GFAP^+^ staining area could also be noted in these animals, but it was weaker as in the ischemia group.

Concerning microglia, a different response was noted. Microglia cells are the resident macrophages of the retina and play an important role in the immune defense against pathogens and as phagocytic cells that remove cellular debris under pathologic conditions. As described before, we noted a significant increase in Iba1^+^ microglia and corresponding *Iba1* mRNA levels after retinal ischemia [57, 58]. Additionally, a strong increase in activated microglia was observed. An early activation of retinal microglia after ischemia has been described before [59]. Microglia cells might be activated in order to phagocyte cell debris that occurred after neuronal cells were damaged through ischemia.

It is well known that retinal ischemia leads to the death of RGCs [9, 60]. Our results indicate that the damage of RGCs after ischemia can be decreased through intravitreal injection of ranibizumab. We detected a significant protection of Brn-3a^+^ and RBPMS^+^ RGCs via immunohistology. While *Brn-3a* mRNA expression was also decreased after ranibizumab treatment, when compared to healthy controls, *Brn-3a* mRNA levels were significantly higher in the ranibizumab group in comparison to the ischemia group. It seems like single injection ranibizumab did already achieve a certain protection of RGCs. Applying ranibizumab three days after ischemia, induction is probably too late, to accomplish a full RGC protection. Further studies should also investigate if the functionality of RGCs is still intact, e.g. via electrophysiology.

Our observation that cholinergic amacrine cells are very sensitive to ischemic damage is in agreement with previous findings by Dijk et al. [61]. They noted a rapid and irreversible reduction of ChAT^+^ amacrine cells. After 48 h, hardly any cholinergic amacrine cells could be observed anymore. In our study, no protective effect of ranibizumab on cholinergic amacrine cells could be noted on mRNA or protein level. Possibly, this retinal cell type is very sensitive to ischemic stress and treatment three days after ischemia is too late, although it was sufficient to observe some neuroprotective effects.

As detected via immunohistology, retinal ischemia induced a decrease in rhodopsin^+^ photoreceptor area. This photoreceptor decrease could be successfully inhibited by ranibizumab treatment. A decreased number of photoreceptors after ischemia has already been described before [57]. This is in accordance with our current observations in the ischemia group, in regard to immunohistology. *Rhodopsin* mRNA levels in our study were neither altered in the ischemic nor in the control groups.

In a RGC-5 (mouse neuronal cells) cell line VEGF protection against oxidative stress is blocked by bevacizumab [62]. Schnichels et al. observed changes in cell viability and proliferation rate in RGC-5 and 661W (mouse photoreceptors) cell lines that were treated with ranibizumab [63].

We noted that application of an anti-VEGF antibody could protect RGCs and photoreceptors, whereas amacrine cells still degenerated. It can be assumed that retinal ischemia causes a VEGF imbalance. In a recent study, diabetic rats were treated with intravitreal anti-VEGF antibody injection. Here, the inhibition of VEGF significantly increased apoptosis in amacrine and bipolar cells in the INL and lead to a slight increase in RGC apoptosis [64]. This finding may suggest that comprised neuronal cells in the diabetic retina could be further affected by treatments. On the other hand, in a NMDA-induced RGC damage animal model, a toxic effect of VEGF inhibitors on RGCs *in vivo* was observed [65]. Different concentrations of bevacizumab, ranibizumab, and pegaptanib did not affect RGC numbers in healthy or NMDA animals, while damaged retinal layers were reported after repeated bevacizumab treatment in rats [66]. In contrast, another study did not note any morphological changes of retinal layers or increased apoptosis after bevacizumab injection in a retinopathy of prematurity mouse model [67]. In clinical studies, possible contradictory effects of VEGF inhibitors are also discussed. In AMD patients treated with ranibizumab a significant retinal nerve fiber layer (RNFL) thinning occurred in the treated eyes, while the cause for this observation is still unclear [68]. Also, the question arises, if this is clinically relevant, since these patients display already a compromised RNFL.

Recently, it could be demonstrated that in a retinal ischemia model neuronal loss was abolished by application of VEGF-A165b [69]. In this study, rats treated with intraocular injection of recombinant human anti-VEGF-A165b antibody, prior to ischemia, exhibited more neurons and reduced apoptosis in both RGCs and inner nuclear layer cells than sham treated animals. A single dose of ranibizumab, after induction of non-arteritic anterior ischemic optic neuropathy (NAION), resulted in no reduction of clinical, electrophysiological, or histologic damage compared to vehicle-treated animals in a non-human primate NAION model [70]. In a rat model of NAION, neither a damaging nor a rescuing effect on RGCs was observed after intravitreal injection of ranibizumab [71]. Also, no benefits of anti-VEGF treatment have been reported in patients with NAION. Nevertheless, in our retinal ischemia model ischemic damage was less prominent after intravitreal treatment with ranibizumab.

### Conclusions

This study demonstrates that intravitreal ranibizumab treatment leads to a certain protection of neuronal cells, like RGCs and photoreceptors, in an ischemia-reperfusion animal model. Ranibizumab seems to rescue some retinal cells against ischemia, while cholinergic amacrine cells could not be saved. These cells seem to be particularly sensitive for ischemic damage. Possibly, an earlier intervention is necessary to protect this amacrine cell type in the ischemia model. Still, ranibizumab could be a potential treatment option for ischemic damage of the retina.
